# Arlington Longitudinal Optimal Healthy Aging Study (ALOHA): A Framework for Aging Research With Limited Resources

**DOI:** 10.1093/geroni/igaf122.1758

**Published:** 2025-12-31

**Authors:** Patricia Heyn, Erin Staker, Mahederemariam Dagne, Catherine Diaz-Asper, Uma Kelekar, Shelly Aboagye, Sara Pappa, J Taylor Harden

**Affiliations:** Marymount University, Arlington, Virginia, United States; Center for Optimal Aging, Arlington, Virginia, United States; Marymount University, Arlington, Virginia, United States; Marymount University, Arlington, Virginia, United States; Marymount University, Arlington, Virginia, United States; Marymount University, Arlington, Virginia, United States; Marymount University, Arlington, Virginia, United States; Marymount University, Fairfax Station, Virginia, United States

## Abstract

As the population of older adults increases, so does the need for sustainable, inclusive, and interdisciplinary aging research, particularly in resource-limited settings. The Arlington Longitudinal Optimal Healthy Aging Study (ALOHA) offers a scalable, community-based framework for tracking health trajectories, cognitive function, and mobility among diverse older adults in Northern Virginia. Led by an interdisciplinary team, ALOHA identifies modifiable risk factors related to aging, such as frailty, cardiovascular health, and cognitive decline. Participants receive personalized results through a “Health Passport”, a toolkit that provides individualized health goals, wellness resources, and behavior change recommendations to support optimal aging. The study focuses on engaging underrepresented populations in a region that mirrors the growing diversity of the United States. ALOHA addresses disparities in access to physical activity, health promotion resources, and preventative care, especially among underserved older adults. Longitudinal data will be used to evaluate participant health trajectories and refine the Health Passport, to expand this model to other communities and research centers. Northern Virginia, recognized nationally for its high community health rankings and demographic diversity, serves as a unique “living laboratory” for developing equitable, accessible, and translational aging interventions. Despite operating with limited funding, ALOHA demonstrates how community partnerships, participant-centered approaches, and interdisciplinary collaboration can drive impactful aging research. This model can inform future policy, enhance healthy aging outcomes, and strengthen research infrastructure in both academic and community settings.

